# Why Is It So Challenging to Measure Residual Stresses ?

**DOI:** 10.1007/s11340-022-00879-x

**Published:** 2022-08-12

**Authors:** G. S. Schajer, M. B. Prime, P. J. Withers

**Affiliations:** 1 University of British Columbia, Vancouver, Canada; 2 Los Alamos National Laboratory, Los Alamos, NM USA; 3 Department of Materials, Henry Royce Institute, Manchester University, Manchester, UK

**Keywords:** Residual Stress, Measurement, Challenges

## Abstract

**Background:**

Residual stresses have a “hidden” character because they exist in a material without the presence of any external loads. They cannot easily be added or subtracted in a quantified manner, as is done when measuring applied stresses, and so are much more challenging to measure.

**Objective:**

The objective here is to identify and describe the various features that make residual stress measurement methods challenging and to consider the ways that these challenges can be addressed in practice.

**Methods:**

Various of the most common residual stress measurements methods are considered and the challenges associated with them are identified and classified.

**Results:**

Five major challenges for residual stress measurements, and the approaches used for their resolution, are identified.

**Conclusions:**

Despite the various challenges that need to be overcome, residual stress measurements can be successfully undertaken in practice. The most significant feature for success is a highly skilled and knowledge practitioner.

## Introduction

Residual stresses have a “hidden” character because they exist in a material in the absence of any external loads [1]. In addition, they also often have a complex 3-dimensional character. They add to the more apparent stresses arising from the applied loads and can lead to serious failures if not adequately accounted for. Residual stresses are introduced throughout all stages of manufacturing and can vary during component life. They are very difficult to predict, and so reliable measurement is essential.

Residual stresses are more complex to measure because they cannot easily be added and/or removed in a quantified manner, as can be done when considering applied stresses. These and other characteristics make residual stress measurement very challenging, so that significant knowledge, judgment and skill are essential to make effective measurements. This paper describes the character and causes of various experimental and theoretical challenges that exist when making residual stress measurements. It is intended as a “commentary” rather than as a review and is aimed towards incoming practitioners and researchers to the field to help guide them on the issues that need to be addressed so as to make effective residual stress measurements.

For applied stress measurements, the unloaded condition is taken as the zero datum. This greatly simplifies measurement procedures and data interpretation. Unfortunately, this convenient circumstance does not exist for residual stress measurements. Residual stresses are “absolute” quantities, in contrast to applied stresses, which are “relative” quantities, (i.e., relative to the unloaded datum). Consequently, all residual stress measurement methods must explicitly include some method for establishing a zero-stress datum [2, 3].

Residual stress measurement methods can be classified broadly into two general classes:

- relaxation (also called “destructive”) methods, in which material is removed and the resulting deformations used to infer the residual stress prior to cutting, and
- non-destructive methods, which rely on measuring some phenomenon that relates directly to the stress, such as the spacing between the atoms, or a secondary effect, such as a change in magnetic or vibration spectra that can be related back to the underlying stress state.

The distinction between the two measurement classes is not as clear as it may seem. In many cases destructive methods do little damage, for example, some methods are described as “semi-destructive” because the damage is slight or can be repaired. Conversely, non-destructive methods often require cutting the sample to gain sufficient access to the location of interest, or to collect reference samples to establish the zero-stress datum. If the need for the creation of a stress-free reference specimen requires the destruction of a second specimen, then the effect is just to transfer the damage elsewhere. Perhaps only when the method completely maintains the integrity of the component can the method truly be called “non-destructive.” Nonetheless, we follow convention and refer to such methods as “non-destructive.”

In the relaxation type of residual stress measurements, some stressed material within the specimen is cut away so as to expose a new stress-free surface that acts as the zero-stress datum of the measurements [4]. The deformation responses from the resulting local redistribution of the residual stresses are measured, from which the originally existing residual stresses are inferred. In this case, the zero-datum challenge is to cut the material in a geometrically accurate way that does not induce additional local residual stresses. In addition, it is often not possible to make deformation measurements directly at the stress-free surface, which provides the zero-stress datum. Thus, the residual stresses originally existing at a specific location need to be inferred from nearby deformation measurements.

For the non-destructive residual stress measurement methods, the typical challenge is to create a “stress-free” reference specimen of the same material to define the zero-stress datum [5]. This is typically done by stress-relieving a separate material specimen, or by grinding some sample material to a fine powder, which is inherently stress-free at the macroscale.

The various relaxation and non-destructive residual stress measurement types each have their advantages and concerns. These features tend to be complementary, with the advantages of one measurement type generally leading to concerns for the other measurement method type. This characteristic can make it desirable to use a combination of measurement methods so as to enable the advantages of each measurement method to compensate for the disadvantage of the others [6]. The relaxation methods are generally applicable to linear-elastic materials. They only require knowledge of the elastic constants, without further material-specific calibrations, however, they necessarily damage the specimen. In contrast, the non-destructive measurement techniques typically keep the specimen intact, but often have limited range of applicability (e.g. are restricted to crystalline, magnetic or Raman active materials) or involve second-order effects that require extensive material-specific calibrations.

A further challenge arises in relaxation type measurements because material continuity causes the relief of a residual stress at a given material location also to create deformations in adjacent locations. The converse is also true, such that the deformation response measured at a given location combines the effects of the relief of the residual stresses in adjacent locations. A sophisticated “inverse” calculation is then required to separate out the individual residual stresses from among the combination of stresses that effect the deformation measurements [7]. In general, this calculation is numerically sensitive, so very accurate measurements are needed to keep noise to acceptable levels.

Applied stress measurements tend to be conceptually straightforward, so a non-specialist practitioner who is conscientious and careful may be expected to be able to produce good results. Because of the zero-stress datum issue, most residual stress measurements tend to be more involved, with much need for careful attention to intricate procedural details. The “learning curve” is relatively long, such that substantial practical experience, knowledge and skill is demanded of a practitioner to achieve reliable and meaningful results. The National Physical Laboratory Measurement Good Practice Guide No.53 [8] remarks on this point explicitly “*Operator skill has been identified as probably the most important factor in achieving a reliable and quality measurement.*” The required operator skill goes significantly beyond having excellent practical capabilities, and includes substantial knowledge and understanding of the principles of the measurement being made. Also essential is a keen appreciation of the warning signs of potentially faulty operation and an understanding of the context and limitations of the results obtained.

In summary, some of the most significant challenges faced when making residual stress measurements are the:

1. need for an absolute zero-stress datum.
2. physical damage that may need to be done to the sample.
3. specific material-dependent calibrations that may be needed.
4. sensitivity to stresses at nearby locations in addition to those at each measurement location.
5. substantial sensitivity to measurement, procedural and analytical imperfections.

These challenges require careful consideration. Consequently, the operator must have substantial knowledge, skill and experience to be able to address them effectively to achieve reliable results. In the following sections, the practical challenges associated with many of the most common residual stress measurement techniques are discussed. The details vary, but generally involve various of the five fundamental challenges listed above.

## Excision Method

**Fig. 1:**
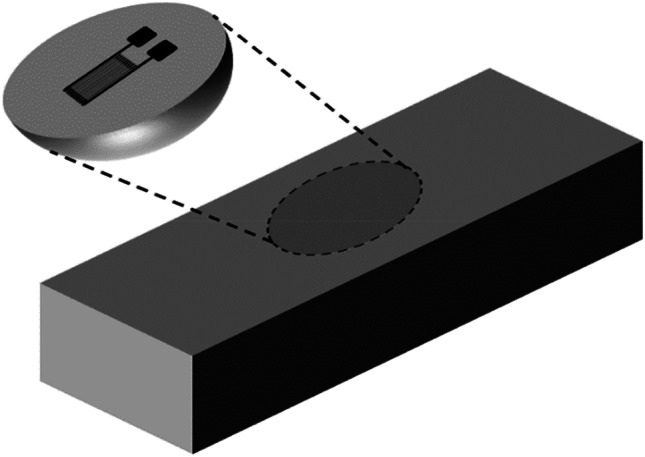
Excision method for measuring residual stresses

The Excision method [9], schematically illustrated in Fig. 1, provides the simplest conceptual example of a relaxation type measurement for evaluating residual stresses. The procedure involves measuring the deformation of a small material fragment, typically using a strain gauge, as it is cut from the bulk of the specimen material. The excised fragment is assumed to be so small that the residual stresses originally within it are fully relieved. In that case the strain gauge will register the full associated strain, from which the surface residual stress can be directly evaluated.

It can be seen that even this conceptually simple residual stress measurement method runs into Challenges 1 and 2 in the above list. The full excision is intended to relieve the local residual stresses completely. However, to achieve this, the excised material fragment needs to be very thin and the cutting process needs to cut cleanly without introducing further residual stresses. Both features are very difficult and time consuming to achieve in practice, thereby including Challenge 5. Thus, despite its conceptual simplicity, the challenges are large, so the Excision Method is rarely used in practice.

## Stress Profiling Methods

**Fig. 2:**
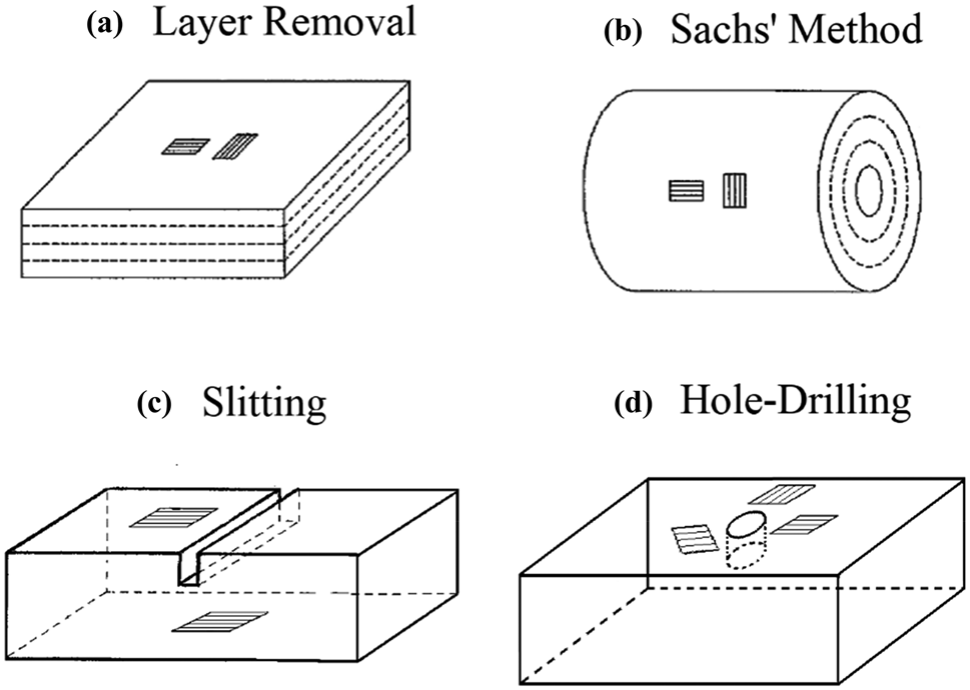
Stress Profiling measurement methods. **(a)** layer removal, **(b)** Sachs’ method, **(c)** slitting, **(d)** hole-drilling

The Stress Profiling methods comprise a large subset of measurement techniques within the category of relaxation measurement methods [7]. These methods all measure a one-dimensional “profile” of one or more stress components. Like all the relaxation measurement methods, they are destructive to various extents. The most common Stress Profiling methods include the Layer Removal method [10], Sachs’ method [11], the Slitting method [12, 13], and the Hole-Drilling method [14]. Figure 2 schematically illustrates these methods. It can be seen that each measurement method is designed specifically for specimens of distinctive geometry and distribution of stresses. Thus, it appears that the various methods are also operationally distinct from each other. This is true in a geometrical sense, but the characteristics of the measurements and of their mathematical analysis are similar for all of them.

The Layer Removal method illustrated in Fig. 2(a) involves attaching strain gauges to the surface of a plate-shaped specimen and measuring the strain changes as layers of stressed material are successively removed from the opposite face. The Sachs’ method illustrated in Fig. 2(b) is an analogous procedure arranged instead to work with a cylindrical specimen. Strain gauges are attached to the outside of the cylinder and the strain changes are measured as layers of stressed material are successively removed from the central area by drilling progressively larger central holes. Alternatively, the arrangement can be reversed and the strain gauges can be attached to the inner surface of a hollow cylinder and strain readings taken as material from the outside surface is progressively removed.

The Slitting method illustrated in Fig. 2(c) involves cutting a narrow slit in a prismatic shaped specimen and measuring the strain responses at nearby strain gauges as the depth of the slit is progressively increased. The Hole-Drilling method illustrated in Fig. 2(d) follows a similar procedure, with strain gauge measurements being made as a hole is progressively drilled into the specimen material. The analogous Ring-Core method [15] (not illustrated in Fig. 2) follows a similar procedure, but with the hole and strain gauge locations reversed; the strain gauges are placed in the central area while the surrounding material is progressively cut away to create a concentric ring.

All the Stress Profiling methods involve making strain measurements while stressed material is progressively removed. At any given step, the relieved strain depends on a weighted integral of the stresses originally existing within the depth of the material cut so far, with a bias towards the stresses nearer the strain gauge(s). In mathematical terms, the relationship can be expressed in the form of a Volterra equation of the first kind [7]

1 $$\varepsilon \left(h\right) = \frac{1}{E} {\int }_{0}^{h} A\left(H,h\right) \sigma \left(H\right) dH$$

where *ε(h)* is the strain measured when the cut depth reaches *h*, and *σ(H)* is the stress originally existing at depth *H*, where *H* ≤ *h*. The kernel function *A(H,h)* describes the strain sensitivity due to the stress at depth *H* within a cut depth *h*. The division by the elastic constant E non-dimensionalizes *A(H,h)* and allows equation (1) to apply to general materials. For layer removal and Sachs’ calculations, *A(H,h)* can be evaluated analytically, for slitting and hole-drilling calculations *A(H,h)* must be evaluated numerically, typically using finite element calculations.

Equation (1) represents an “inverse problem” because the quantity to be evaluated, the stress profile *σ(H)*, is contained within the integral term on the right side. This is the reverse of the conventional situation where the quantity to be evaluated is alone on the left side, and so may be evaluated explicitly. This reversal substantially complicates the required calculations and causes the results to be very sensitive to measurement noise. The effect is like a noise amplifier, where small deviations in the measured strains cause proportionally much larger deviations in the computed stresses (Challenge 5). For the Slitting and Hole-Drilling methods, the effect is made worse by the smaller strain reliefs that are measured relative to the Excision method.

The degree of noise sensitivity in the residual stress evaluation is controlled by the kernel function *A(H,h)* in equation (1), which in turn is controlled by the physical arrangement of the residual stress measurement. As a general rule-of-thumb, the noise sensitivity depends primarily on the distance between the target stress location and the corresponding measurement location, the greater the distance, the higher the noise sensitivity. Thus, for Hole-Drilling, where the strain gauges are mounted on the surface, the noise sensitivity for near-surface stress evaluation is relatively moderate, but steadily increases for deeper stress evaluations. A similar behavior occurs with Slitting measurements using a top-surface mounted strain gauge. In addition, moving the strain gauge closer to the slit position gives focus more strongly on the near-surface stresses, but at the expense of increased noise sensitivity for deeper stress evaluations.

A different behavior occurs in the Slitting Method when using a back-surface strain gauge. Good stress sensitivity is maintained over a wide range of interior stresses from about 5% to at least 95% of the specimen thickness [13, 16, 17]. This desirable response occurs because the slit cutting causes a bending effect in the remaining material ligament below the slit. The associated bending stress changes are maximum at the extreme locations within the remaining material ligament (at the bottom of the slit and at the back surface). This bending effect transmits the effect of the relief of the stresses at the bottom of the slit to the back surface, where they are measured by the back-surface strain gauge. By comparison, top-surface strain gauges have very limited stress range and are now rarely used.

For all Stress Profiling methods, increasing the number and fineness of the cut depth steps made during the measurement has the beneficial effect of increasing the data content of the measurement and provides the opportunity for increase in spatial resolution of the evaluated stress profile. However, sensitivity to measurement noise also substantially increases. This is a typical characteristic of the solution from an inverse equation such as equation (1). Conversely, fewer but larger cut depth steps reduces the measurement noise sensitivity, but at the expense of data content and spatial resolution. A compromise can be achieved using smoothing, typically by using the Tikhonov regularization technique [7]. A large number of cut depth steps is used to increase data content, but with regularization used during the mathematical solution of equation (1) to moderate the associated noise sensitivity. By carefully limiting the amount of regularization used to the minimum, substantial noise reduction can be achieved with only modest loss of spatial resolution. Increased regularization can further decrease noise sensitivity, but at the expense of reduced spatial resolution.

The greater level of procedural complexity of the Stress Profiling methods relative to the Excision method causes them to run into more of the listed measurement challenges. In common with all relaxation type measurements, the “zero datum” in Challenge 1 is achieved by exposing stress-free surfaces within the material. Very careful cutting is required to produce geometrically accurate surfaces that relieve the original residual stresses without inducing additional stresses. This is done through the use of sharp cutting tools, small cutting depths and the minimization of material heating. For Hole-Drilling, orbital cutting is commonly used, while for Slitting, wire EDM cutting is a very effective choice when available [18]. Specimen physical damage, mentioned in Challenge 2, is an unavoidable issue among all relaxation methods. The Hole-Drilling method is a potential exception because the damage is modest and possibly tolerable. Thus, the method is sometimes described as “semi destructive”.

The challenge of solving the inverse problem, described in Challenge 4, is also a common feature among the Stress Profiling methods, as exemplified by equation (1). Thus, an inverse calculation is required to determine the residual stress profile from the measured strain data. This calculation tends to amplify the effects of measurement noise, particularly when the target stresses are relatively far from the measurement location(s). This feature brings Challenge 5 into play, particularly when greater spatial resolution of stresses is sought, making scrupulous measurement technique and careful control of regularization amount essential for all measurement types.

The “relaxations” that occur when using relaxation measurement techniques are assumed elastic, so for given elastic constants the calibration data are universal and apply to all linear elastic materials. Thus, beyond a knowledge of the material elastic constants, specific material-dependent calibrations such as mentioned in Challenge 3 are not required. For the layer removal and Sachs’ methods the kernel functions are known analytically. For the Slitting and Hole-Drilling methods, they are determined numerically, typically using finite element calculations. In the latter two cases, stress concentrations at the cut location can cause the material response to go out of the elastic range for residual stresses near the yield point, in which case the residual stress calculations based on elastic response can be in error [19].

## Contour Method

**Fig. 3:**
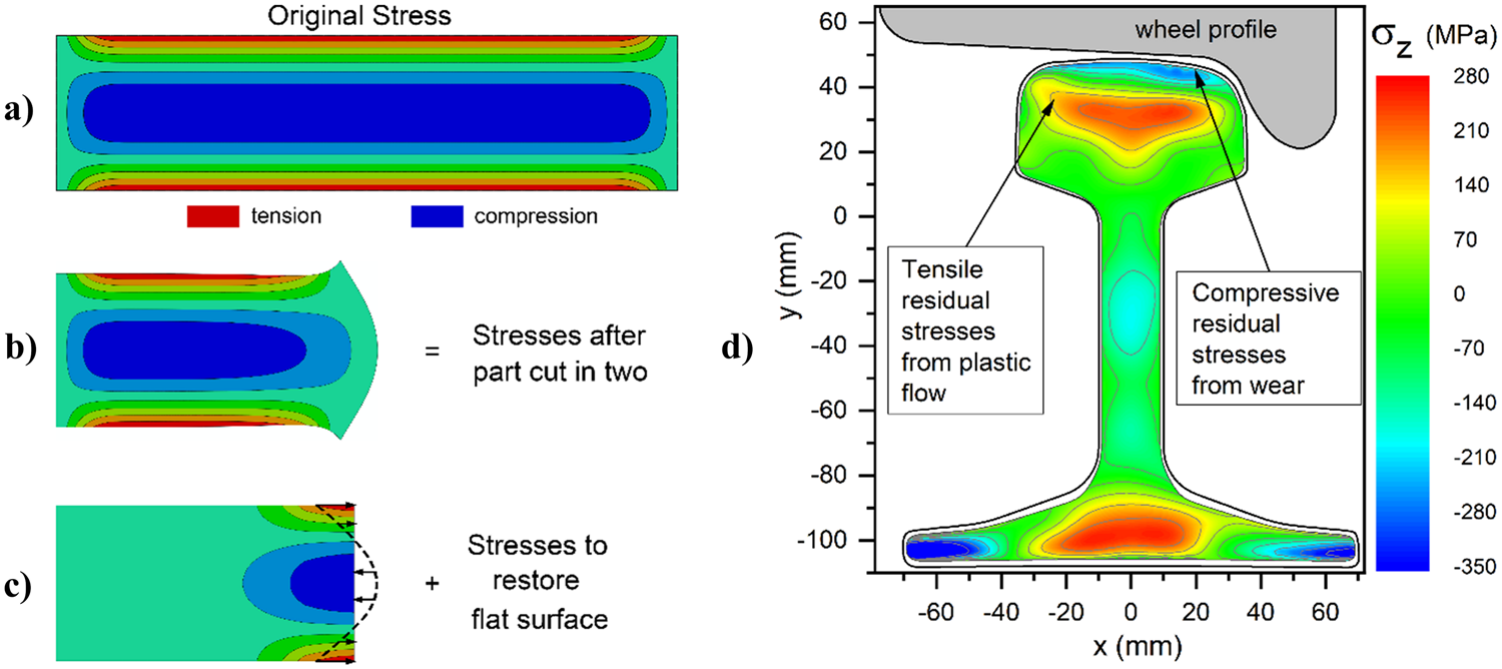
Contour Method. **(a)** Original stresses, **(b)** Stress-free after cutting, **(c)** Stresses to restore flat surface, **(d)** Measured stress profile of a worn railway rail

The Contour Method [20], illustrated in Fig. 3, is notable among the relaxation type measurement techniques in that it can provide a detailed two-dimensional map of the normal residual stresses acting on a plane within the specimen. The procedure involves cutting through the specimen cross-section using wire Electro-Discharge Machining (EDM), and measuring the surface height maps of the cut surfaces using a coordinate measuring machine or a laser profilometer. The residual stresses shown in Fig. 3(a) are released by the cut and cause the material surface to deform (retract inwards for tensile stresses, bulge outwards for compressive stresses), as shown in Fig. 3(b). The originally existing residual stresses normal to the cut can be evaluated from finite element calculations by determining the stresses required to return the deformed surface shape to a flat plane, as shown in Fig. 3(c). To avoid any asymmetry effects, it is important to measure the surfaces on both sides of the cut and to use the average surface height map. It is also possible to make further cuts on perpendicular planes to get maps of the normal residual stresses on those planes [21]. Figure 3(d) shows an example measurement of the axial residual stress profile within the cross-section of a worn railway rail [22].

The Contour Method is remarkable for its conceptual simplicity and 2-D measurement capacity. However, in common with other residual stress measurement methods, substantial challenges must be addressed to get reliable results. The “zero-stress datum” in Challenge 1 is achieved by exposing the stress-free surface shown in Fig. 3(b), and measurements are taken directly on this surface. In practical measurements, it is convenient to define a reference plane as the best-fit average of the cut plane. This arrangement keeps the surface shape measurements as small numbers. However, the exact positioning of this reference plane is not critical because the subsequent finite element analysis of the measured data automatically rotates and translates the reference plane so as to satisfy force and moment equilibrium [22].

In practice, EDM cutting limitations, or the presence of the residual stresses, can cause minor deviations in the through-thickness cut made when implementing the Contour method. Although these deviations typically are small, they can be significant because the target surface deformations to be measured for the Contour method are also small and can be comparable in size. Consequently, it is usual to measure both cut surfaces and to use the average of the two measured shapes when computing the residual stress distribution. This effectively removes the effect of minor trajectory errors in the cut shape, but not of yielding effects that may occur in highly stressed materials [23, 24]. For the averaging procedure to work effectively, the cut width must be kept uniform to μm levels. This can be achieved by using a wire EDM machine for the cutting; it has the further important feature of inducing minimal additional residual stresses [18]. A high-precision coordinate measuring machine or laser profilometer is required to achieve the required surface shape measurement resolution. Challenge 5 is a substantial issue with Contour measurements; very sophisticated equipment run by skilled operatives is essential for both cutting and measurement steps to get successful results.

Cross-sectional slicing of the specimen causes major damage, so the Contour Method is clearly subject to Challenge 2. It is also subject to Challenge 4 insofar as the measured displacement at each surface point depends on the originally existing residual stresses both at that point and at the neighboring surface points. There is also a minor sensitivity to out-of-plane shear stresses. Because deformations are measured directly at the location of stress relaxation, the associated inverse problem is much simpler than equation (1) and is solved within the finite element calculation that makes the step between Fig. 3(b, c). Despite the simpler inverse problem, noise amplification is an issue as with the Stress Profiling methods, so the raw data must be smoothed, and the resulting uncertainties estimated [25]. As with the other relaxation type methods, the responses measured within the Contour method are assumed elastic, so beyond a knowledge of the material elastic constants, specific material-dependent calibrations such as described in Challenge 3 are not required.

## Laboratory X-ray Method

**Fig. 4:**
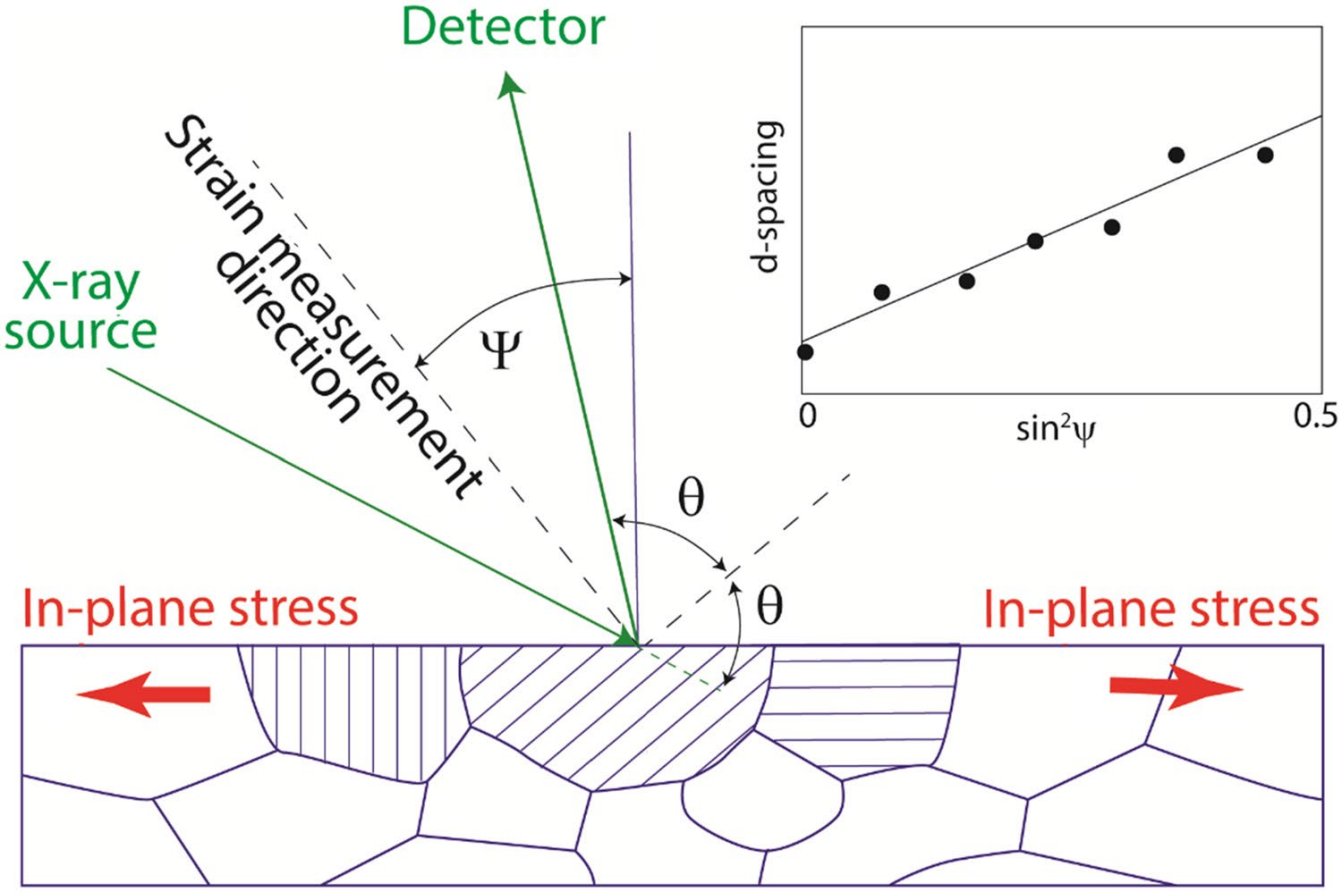
Diffraction of X-rays from near surface crystal grains oriented for diffraction from the (hkl) crystalline lattice planes

The Laboratory X-ray method [26] is a “non-destructive” diffraction-based technique [5]. It is effective with crystalline materials and uses the atomic spacing between specific crystal “(hkl)” lattice planes as an elastic “strain gauge.” The atomic spacings between these planes change according to Hooke’s Law and the associated strains can be identified though the changes in diffraction angles of X-rays incident on the material surface. Figure 4 schematically shows X-ray radiation of wavelength *λ* incident on a crystal lattice of atomic plane spacing *d*_(hkl)_, where the notation “(hkl)” identifies that specific crystal plane that is being used. The plane spacing can be determined from the angle through which X-rays are diffracted according to Bragg’s Law:

2 $$\mathrm{sin}{\theta }_{(hkl)} = \frac{\lambda }{2{d}_{(hkl)}}$$

where *θ*_*(hkl)*_ is the corresponding X-ray diffraction angle. Laboratory X-ray sources generally exploit characteristic X-ray wavelengths from Cr (wavelength of 0.229 nm) to Mo (0.071 nm), which are similar to the spacing between planes of atoms and so scatter to large diffraction angles. The low penetration depth (typically no more than tens of microns) of these moderate energy laboratory X-rays mean that they can only probe the near surface state non-destructively. In most cases this means that it is not possible to measure the in-plane strain directly, but instead it must be evaluated by inclining the measurement direction so as to sample a component of the in-plane strain. Measurement of the residual stress state is thus done by tilting the sample and measuring the lattice spacings using a range of incident X-ray angles, most commonly using the “sine-squared psi” method [27], although the “cos alpha” method [28] is increasingly applied when using area detectors. Psi and alpha are angles describing the spatial orientation of the diffracted X-ray response.

Key to the measurement of strain by all diffraction methods is the determination of the unstrained atomic spacing *d*_*0*(hkl)_. This is needed to provide the zero datum for the measurements when converting the lattice spacings into the elastic strain ε_(hkl)_ = *(d*_*(hkl)*_*-d*_*0*(hkl)_*)/d*_*(hkl)*_. While the out-of-plane strain needs not be zero, the fact that the out-of-plane stress is zero means that the out-of-plane strain is usually close enough to being strain-free [26]). Thus, Challenge 1 is often tractable.

It is important to note that diffraction measurements sample a very specific subset of the material, i.e., those grains with crystal plane (hkl) oriented in a particular direction relative to the incident radiation beam as shown in Fig. 4. However, the “micro-stresses” within a single crystal phase may not always be representative of the “macro-stresses” averaged within the material as a whole, and may even show non-zero out-of-plane stresses. Thus, great care needs to be exercised to choose an incident radiation direction that will provide a faithful representation of the overall macro-stress. In addition, the radiation spot size must be large enough to span a significant amount of material, but also small enough to fit within geometrical features of the specimen. More specialized procedures are required when working with multi-phase and non-isotropic materials, causing Challenge 5 to become even greater. Signs of problems are significant differences in results from multiple reflections, irregular variations in peak height or integrated intensity and FWHM (full width at half maximum) with psi-tilt for various reflections as well as non-linear sine-squared psi responses [29]. Practical guides [30] have been produced to assist practitioners, who must have informed skill and understanding of how to interpret X-ray results appropriately.

As with the Stress Profiling methods, there is a tradeoff between spatial resolution and sensitivity to measurement noise (Challenge 5). For diffraction methods, sensitivity to measurement noise can be reduced by increasing the volume of material that is being sampled during the measurement (the “interrogation” or “sampling” volume). The greater the sampling volume, the greater the number of grains being sampled and hence measurement noise is reduced. However, the greater sampling volume reduces the available spatial resolution of the resulting stress evaluation.

In order to make measurements at depth, it is necessary to remove material from the surface so that the method becomes destructive. Because the stress state measured at the surface may be affected by scratches or other surface effects, it is often recommended to remove a few tens of microns of material from the surface by electro-polishing. If a map of the variation in stress with depth (up to a few millimeters are practical) is desired, then it is important to note that the required surface material removal may redistribute the out-of-plane stress components. The effect of this redistribution can be taken into account mathematically [31].

The penetration of laboratory X-rays is limited by the low energies (~ 10 keV) associated with the characteristic radiation emitted by the X-ray target. Synchrotron X-ray sources can provide very intense X-ray beams having energies up to hundreds of keV [32]. The basic governing equations are the same as for Laboratory X-ray diffraction, but the greater penetration means that the region sampled by the x-ray beam may have a triaxial stress state such that no direction can generally be assumed to be stress free, and so the determination of the strain-free lattice spacing is a major challenge [33]. This issue (Challenge 1) is shared with the Neutron Diffraction method, described in more detail in the next section.

## Neutron Diffraction Method

The Neutron Diffraction method [34, 35] is also a diffraction-based measurement technique. Neutrons are produced by nuclear reactors in a continuous stream, and in pulses by spallation sources. As with the X-ray method, it uses the plane spacing within the crystalline structure of a metal as a “strain gauge”, and evaluates that spacing by diffraction measurements and the use of Bragg’s Law, equation (2). However, the practical procedural details are substantially different. Because neutrons are uncharged particles, their interaction with the specimen material is much less than with X-rays, so they are able to penetrate several tens and even hundreds of mm. This penetration depth of neutrons gives the Neutron Diffraction method the important ability to probe the interior of moderate to large size specimens. In contrast, Laboratory X-ray measurements can penetrate only a few μm, and give only surface stress information.

**Fig. 5:**
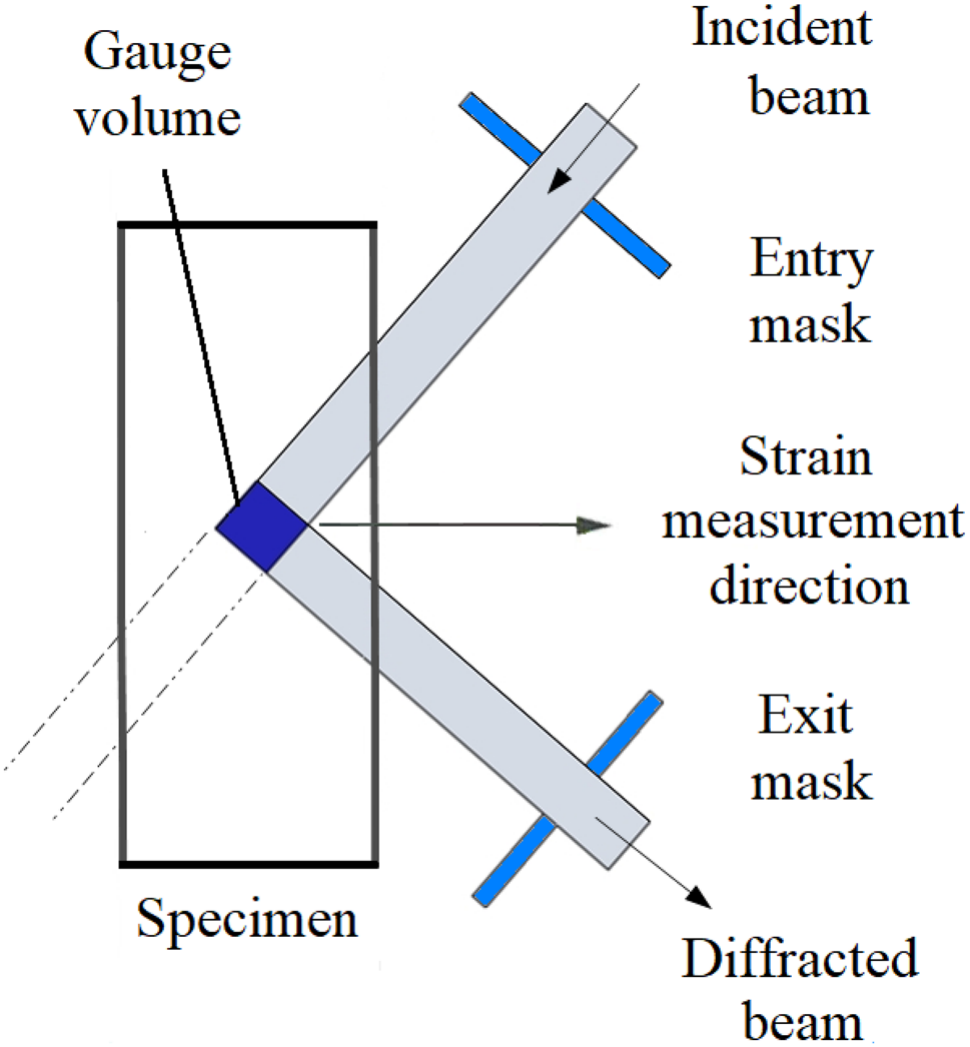
Schematic arrangement of a residual stress measurement by neutron diffraction

Figure 5 shows a schematic diagram of a typical Neutron Diffraction measurement arrangement. A beam of moderated neutrons from a spallation or reactor source is directed at the specimen along a path whose location is defined by the position of an entry mask, typically made of cadmium. The neutron beam is diffracted within the test specimen and exits through a second cadmium mask with fixed position relative to the entry mask. The intersection of the paths of the entry and exit neutron beams defines the “gauge volume” within which the diffraction measurements occur, typically comprising several mm^3^. The specimen can be moved through the gauge volume so that the strain field within the specimen can be scanned in a systematic manner.

While conceptually straightforward and with attractive non-destructive interior stress measurement capabilities, the Neutron Diffraction method is challenging to implement in practice [35]. Most significantly, it requires a source of thermal neutrons, which is available only within a major nuclear facility. The accurate location of the gauge volume deep within a complex test specimen often requires a highly skilled operator. Further, the fact that residual stresses deep within a body can be triaxial, such that the zero out-of-plane surface stress condition invoked in surface X-ray stress measurement not available, means that Challenge 1 becomes a major issue. Indeed this is exacerbated by the fact that the strain free crystal spacing, *d*_*0,*_ often varies with position through the sample because of differences in thermal histories and inhomogeneous chemical composition [36]. Usually, the zero strain datum must be established using separate stress-free specimens of the same material, typically in the form of a powder, comb or small cube [37]. Since changes in lattice spacing arising from thermal expansion cannot be distinguished from mechanical strains, it is also essential to ensure that the temperatures of the test and stress-free specimens are kept identical.

## Other Non-destructive Methods

The relaxation and diffraction methods described above are effective for a wide range of materials. They have the advantage of involving a “primary” relationship, Hooke’s Law, to connect the measurements to the local stress. There are several other methods that infer the state of stress from “secondary” relationships, for example:

- Ultrasonic methods
- Magnetic (Barkhausen) methods

The Ultrasonic methods [38, 39] depend on the acousto-elastic effect, where the presence of stress causes small changes in the speed of sound transmission through metallic materials. The Magnetic methods [40] depend on the effect of stresses on material magnetic properties, usually the acoustic Barkhausen noise that is generated due to magnetostriction and magnetic domain orientation change in a ferromagnetic material as its magnetization state is being cycled.

The great advantage of many of these non-destructive methods is that they are relatively cheap and rapid to apply. Once set up, they can be operated automatically, and so are well suited to repeated or routine applications such as industrial quality control testing. However, their disadvantage is that they do not have a convenient zero datum (Challenge 1), so initialization with stress-free specimens is needed. In addition, the methods are very material specific and typically require individualized, time-consuming calibrations to fit the target test material (Challenge 3). Even small changes in specimen material can alter calibration settings quite substantially, so the methods are limited to quality control testing of large batches of nominally similar material. Setup and calibration typically require experienced operatives to achieve reliable results.

## Conclusions

The “hidden” nature of residual stresses causes their evaluation procedures to be much more challenging than those for the relative measurements of applied stresses. These challenges can manifest in several ways:

1. The need for an absolute zero-stress datum. This is often difficult to achieve in practice. For relaxation type measurements, highly accurate, stress-free cutting is essential. For other methods, stress-free reference specimens must usually be prepared which are identical in state to the residually stressed location of interest.
2. The sample may need to be physically damaged in order to make the measurements. Such damage occurs with all relaxation type measurements, and sometimes also with diffractive measurements. This issue is a particular concern when working with non-disposable specimens, when the part is still in service, or when multiple measurements are desired on the same specimen.
3. Specific material-dependent calibrations may be needed. This issue arises to some extent with all measurement methods, but is a particular challenge with the “Other Non-destructive Methods”, where small variations in material composition or preparation can require substantial changes in the calibration.
4. Sensitivity to stresses at nearby locations in addition to those at the measurement location. This issue typically creates the need for “inverse” calculations.
5. Substantial sensitivity to measurement and procedural imperfections. Consequently, a very high standard of measurement and procedural precision is required to achieve effective results.

The combination of all of these challenges causes residual stress measurements to be highly sensitive to adverse influences. Thus, residual stress measurements tend to have wider error bars than analogous applied stress measurements. This does not mean that residual stress measurements are inferior to applied stress measurements. Actually, one may argue the opposite because residual stress measurements generally demand methods of higher sophistication.

The above five challenges place substantial demands on the operator, thus substantial knowledge, skill and experience are required. The community of residual stress practitioners is a relatively small one within the world of experimental mechanics. However, it is quite active and is specialized in the various residual stress measurement methodologies. This specialization is required by the high level of detailed knowledge and experience to be able to address all five challenges listed above. Thus, as noted by the UK National Physical Laboratory [8], operator skill is indeed a very important factor. The learning curve is somewhat long, but fortunately, various guides have been produced to help practitioners make reliable measurements e.g., [2, 6, 8, 41–44]. In addition, several specialized residual stress conferences take place regularly to share emerging best practices and to offer training courses to new practitioners.
